# RBFOX3/NeuN is dispensable for visual function

**DOI:** 10.1371/journal.pone.0192355

**Published:** 2018-02-05

**Authors:** Yi-Sian Lin, Kuan-Ting Kuo, Shih-Kuo Chen, Hsien-Sung Huang

**Affiliations:** 1 Graduate Institute of Brain and Mind Sciences, College of Medicine, National Taiwan University, Taipei, Taiwan; 2 Department of Pathology, College of Medicine, National Taiwan University, Taipei, Taiwan; 3 Department of Life Science, College of Life Science, National Taiwan University, Taipei, Taiwan; 4 Neurodevelopmental Club in Taiwan, Taipei, Taiwan; National Eye Centre, UNITED STATES

## Abstract

RBFOX3/NeuN is a neuronal splicing regulator involved in neural circuitry balance, as well as neurogenesis and synaptogenesis. *Rbfox3* is expressed in neurons; however, in the retina, expression is restricted to cells in the ganglion cell layer and some cells of the inner nuclear layer. *Rbfox3* is expressed in a layer-specific manner in the retina, which implies a functional role, however, the role of RBFOX3 in the retina is unknown. *Rbfox3* homozygous knockout (*Rbfox3*^*-/-*^) mice exhibit deficits in visual learning; therefore, understanding the role of RBFOX3 in the retina is critical for interpreting behavioral results. We found *Rbfox3* expression was developmentally regulated in the retina and specifically expressed in ganglion cells, amacrine cells and horizontal cells of the retina. We demonstrate deletion of *Rbfox3* resulted in a reduction in the thickness of the inner plexiform layer of the retina, where synapses are formed. Number of ganglion cells and amacrine cells is normal with loss of *Rbfox3*. Innervation of retinal ganglion cells into their targeted brain regions is normal in *Rbfox3*^*-/-*^ mice. Importantly, *Rbfox3*^*-/-*^ mice displayed normal non-image and image forming functions. Taken together, our results suggest RBFOX3 is dispensable for visual function.

## Introduction

RBFOX3 (RNA binding protein, fox-1 homolog (C. elegans) 3), a neuronal splicing regulator also known as NeuN, is a well-known marker of mature neurons [1]. RBFOX3 regulates neuronal differentiation by alternative splicing of *Numb* [2]. Our previous work showed that RBFOX3 is required for hippocampal circuit balance and function in addition to regulating neurogenesis and synaptogenesis [3, 4]. Deletion of *Rbfox3* contributes to increased expression of plasticity genes such as *Erg4* and *Arc* and increased spine density and dendritic complexity in the brain [4]. Moreover, dysfunctional RBFOX3 was identified in persons with neurodevelopmental disorders such as epilepsy, neurocognitive disorders, developmental delay and speech disorders, autism, and sleep disorders [5–9]. In the retina, RBFOX3 is specifically expressed in the cells of the ganglion cell layer and some cells in the inner nuclear layer [10–12]. Layer-specific expression of *Rbfox3* in the retina suggests a functional role. However, the role of RBFOX3 in the retina and visual function is unknown. Our previous work showed deficits of visual learning in *Rbfox3*^*-/-*^ mice [4], therefore, understanding the role of RBFOX3 in the retina and visual function is critical for interpreting behavioral results in *Rbfox3*^*-/-*^ mice.

The visual system provides a means for examining visual perception and non-image forming function, such as pupillary light reflex and circadian rhythm, through two parallel visual pathways. The retina is the first stop in the visual system pathway to transduce light stimuli and send visual information to distinct brain regions. Retinal ganglion cells are responsible for the transmission of light information to downstream brain regions, while amacrine cells and horizontal cells provide lateral computation for visual perception. Despite some key transcription factors that influence the development of retina neurons, molecules critical for the function of retinal ganglion cells have not been fully explored.

Here we use *Rbfox3* homozygous knockout (*Rbfox3*^*-/-*^) mice as a model to investigate the role of RBFOX3 in the retina and visual function. We found that in the retina, the expression of *Rbfox3* was developmentally regulated and expressed exclusively in the ganglion cells, amacrine cells and horizontal cells of the retina. Deletion of *Rbfox3* reduced the thickness of the inner plexiform layer of the retina, where synapses are formed. Number of ganglion cells and amacrine cells is normal with loss of *Rbfox3*. Moreover, we found no difference between *Rbfox3*^*-/-*^ mice and their wild-type counterparts in the innervation of axons of ganglion cells to their downstream brain regions. Non-image-forming function and image-forming function was normal for *Rbfox3*^*-/-*^ mice when examined by the pupillary light reflex test and optomotor response test, respectively. Our results suggest deletion of *Rbfox3* results in minor changes in retinal morphology but normal innervation of retinal ganglion cells, and normal visual function is maintained.

## Materials and methods

### Mice

Detailed information regarding the *Rbfox3*^*-/-*^ mice and genotyping information has been previously described [4]. Mice were group-housed in ventilated cages, given food (PicoLab^®^ Rodent Diet 20, 5053) and water *ad libitum* and maintained on a 12-h light/dark cycle (lights off at 8 pm). Male *Rbfox3* heterozygous knockout mice were mated to female *Rbfox3* heterozygous knockout mice to obtain *Rbfox3* homozygous knockout mice and littermate control wild-type mice. The National Taiwan University College of Medicine and College of Public Health Institutional Animal Care and Use Committee (IACUC) approved all procedures. The approval number is 20140449. All experiments in this study were performed in accordance with the approved guidelines.

### Western blot analysis

Retinas were dissected from wild-type mice at ages postnatal day 0 (P0) to 49 (P49) and from *Rbfox3*^*-/-*^ mice at age P49. Western blot analysis was performed as described previously[4]. Total retinal protein lysates (80 μg) were used. Primary antibodies used were mouse anti-NeuN (1:1,000, Millipore, MAB377) to detect RBFOX3 protein, mouse anti-ACTIN (1:5,000, Sigma, A1978), and rabbit anti-GAPDH (1:6,000, Genetex, GTX118619). Blots were incubated with primary antibodies overnight at 4°C and detected by incubating with their corresponding secondary antibodies at room temperature for 1 h: IRDye680 donkey anti-mouse IgG (H+L) (1:15,000, LI-COR, 926–68072) and IRDye800CW donkey anti-rabbit IgG (H+L) (1:15,000, Li-COR, 926–32213). GAPDH is more stable during development, and therefore for assessment of RBFOX3 from P0 to P49 protein was normalized to levels of GAPDH detected in each sample. For P49 studies protein was normalized to levels of ACTIN detected in each sample. BLUeye prestained protein ladder (GeneDireX, PM007-0500) was used to estimate the size of protein.

### Immunofluorescence staining

Mice (P49) were deeply anesthetized with isoflurane and perfused transcardially with 4% paraformaldehyde in 0.1 M phosphate buffer (PB), pH 7.4. Eyeballs were enucleated and post-fixed for 30 min, and cryoprotected with 30% sucrose in 0.01 M PB for 1 h, then embedded in OCT compound. Cross sections (16 μm) were cut on a cryostat (CM3050 S, Leica), mounted on glass slides, permeabilized with 0.3% Triton X-100 in 1X PBS for 30 min, and blocked with 5% goat or donkey serum for 1 h at room temperature. Sections were incubated with primary antibodies as follows: anti-NeuN (1:500, Millipore, MAB377 & ABN78), to detect RBFOX3 protein, anti-Calbindin (1:1,000, abcam, ab11426, kindly provided by Dr. Yi-Shuian Huang), anti-BRN3A (1:200, Santa Cruz, sc-8429), anti-Calretinin (1:200, Swant, 6B3), anti-TH (1:200, Millipore, MAB318), anti-NF-L (1:500, Novus, NB300-131) and anti-CHAT (1:100, Millipore, AB144P), overnight at 4°C followed by 2 h incubation at room temperature with corresponding secondary antibodies: Alexa Fluor® 488 Goat anti-mouse IgG (H+L) (1:500, Invitrogen, A11001), Alexa Fluor® 488 Goat anti-mouse IgG1 (γ1) (1:500, Invitrogen, A21121), Alexa Fluor® 546 Goat anti-rabbit IgG (H+L) (1:500, Invitrogen, A11010), Alexa Fluor® 594 Goat anti-mouse IgG_2b_ (1:500, Invitrogen, A21145), and Alexa Fluor® 594 Chicken anti-goat IgG (H+L) (1:500, Invitrogen, A21468). Sections were counterstained with DAPI (1:10,000, Invitrogen, D-1306). For whole-mount retinal preparation, the retina was detached from the retinal pigment epithelium. Four radial cuts were made to flatten the retina. Retinas were then post-fixed again for 2 h and blocked with 5% goat or donkey serum for 2 h, incubated with primary antibodies as described above, for 4 days at 4°C, followed by 10 x 6 min wash, 2 h incubation with corresponding secondary antibodies described above, and 10 x 6 min wash. Retinas were then flattened and mounted on glass slides with the GCL side up.

### Hematoxylin and eosin staining

Mice (P49) were deeply anesthetized with isoflurane and their eyes were then enucleated and post-fixed in 10% formalin for 36 h. Sections (5 μm) were cut through the center of the eye (determined by the presence of the optic nerve head) and stained with hematoxylin and eosin (H&E). Light microscopy images were used to analyze retinal layer thickness. Each layer thickness was normalized to total retinal thickness.

### Imaging and quantification

Immunofluorescence images were acquired using a Zeiss LSM780 confocal microscope (Carl Zeiss, Taiwan). For cross-section stained retinas, confocal z-stacks were obtained. For quantifying BRN3A-positive, Calbindin-positive, and CHAT-positive cells in whole mount retina, images of two random areas (size 212.55 x 212.55 μm) of each retinal lobe were obtained 1 mm from the optic nerve head. Cell number of each image was counted manually. For H&E stained retinas, images were acquired using a Zeiss Axio Imager M2 microscope with a 40X/0.75 NA objective to analyze retinal layer thickness of central, middle, and peripheral retina. Each layer thickness was normalized to total retinal thickness.

### Cholera toxin B subunit injection

Mice (P49) were anesthetized with avertin. The right eye was injected intravitreally with 1 μL of cholera toxin B subunit (CTB) conjugated with Alexa Flour 568 (Biotium, 00071) and the left eye was injected intravitreally with 1 μL of CTB conjugated with Alexa Flour 488 (Biotium, 00070). Mice were perfused three days after injection. Brains were removed and post-fixed overnight at 4°C, cryoprotected with 30% sucrose in 0.01 M PB, and embedded in OCT compound. Cross sections (40 μm) were cut from the optic chiasma to the cerebellum. Brain slices were counterstained with DAPI (1:10,000, Invitrogen, D-1306) and mounted on glass slides. Retinal ganglion cell (RGC) axon terminals in the target brain regions were visualized under a Zeiss Axio Imager M2 microscope. No animal mortality occurred before planned sacrifice because Cholera toxin B subunit does not contain the catalytic subunit of the toxin. Immediately after surgery, animals were put under heat lamp to keep them warm before the effect of anesthetic drug wear off. We then put them back to home cage 1–2 hour after they regained activity. We checked their body weight every day for 3 days after injection.

### Behavioral measures

All behavioral assessments were performed with the same group of male wild-type or *Rbfox3*^*−/−*^ mice (9 wild-type and 6 *Rbfox3*^*−/−*^ mice for the PLR test; 9 wild-type and 7 *Rbfox3*^*−/−*^ mice for the optomotor response test). Mice for the PLR test were derived from 8 litters and mice from the optomotor response test were derived from 10 litters. Testing began when mice were 8–15 weeks of age.

#### Pupillary light reflex (PLR) test

The PLR method was modified from Chen *et al*., 2011[13]. Mice were maintained under a 12:12 light-dark cycle prior to testing. Experiments were performed on three consecutive days, at the same time each day. Prior to each experiment, all mice were dark-adapted for at least 1 h. While one eye received light stimulation with a 1500-, 150-, or 15-lx handmade white light-emitting-diode device, an IR-sensitive CCD camera (STD-JE100; Sony) was used to record from the opposite eye for 30 s. A 850 nm infrared illuminator (KOODYZ) was applied to enhance dark sight recording. Total area of the pupil was measured approximately 25–30 s after initiation of the stimulus using ImageJ. The area of pupil was normalized to the area of the whole eye to minimize variation caused by head movements. The percentage of pupillary constriction was calculated as the percentage of final pupil size relative to the pupil size before light stimulation.

#### Optomotor response test

An optomotor system was handmade and modified based on the method described in Prusky *et al*., 2004 and Douglas *et al*., 2005 [14, 15]. To perform the visual acuity test, mice were placed on a platform, which measured 6 cm in diameter, 15 cm above the floor, and surrounded by four 24” LED monitors (Acer P246HL). The monitors faced the windows of a box made of corrugated board (56 x 56 x 30 cm). Vertical grating stimuli were presented synchronously on the monitors under control of a program written in PsychoPy v1.8 (Python) (S1 Code). To assess mouse visual acuity, rotating sinusoidal grating stimuli with spatial frequencies ranging from 0.05 to 1.0 cyc/deg were presented at a constant speed (12 deg/s), in either clockwise or counterclockwise direction. To accelerate the experimental procedure, random white or gray stimulation was displayed on the screens to draw attention of the mice back to the monitor. A CCD camera (STD-JE100; Sony) was mounted on the lid of the box in order to record behavior inside the box, and a home camera (YI technology) was mounted on the corner of the box to provide viewing from a different perspective. Tracking response was initiated if the mouse was able to see the motion on the monitor, which was confirmed when the direction and speed of the head and neck movements of the mouse were in agreement with the clockwise and counter-clockwise of the rotating stimuli. A researcher observed the tracking behaviors in real-time via the laptop screen and smartphone connected to the CCD camera and home camera, respectively. Threshold of visual acuity of each mouse was obtained when the mouse was no longer tracking.

### Statistical analysis

All data are presented as means ± standard error of the mean (s.e.m.) with sample sizes (n) shown in figures or stated in the text. Statistical analyses were performed using SigmaPlot 11 (Systat Software). Normality tests (Shapiro-Wilk) and equal variance tests were run and passed (P > 0.05) before parametric statistical analyses were run. Non-parametric statistical analyses were performed if normality and equal variance tests were not passed (*P* < 0.05).

## Results

### Retinal *Rbfox3* expression is developmentally-regulated and layer-specific

**Fig 1:**
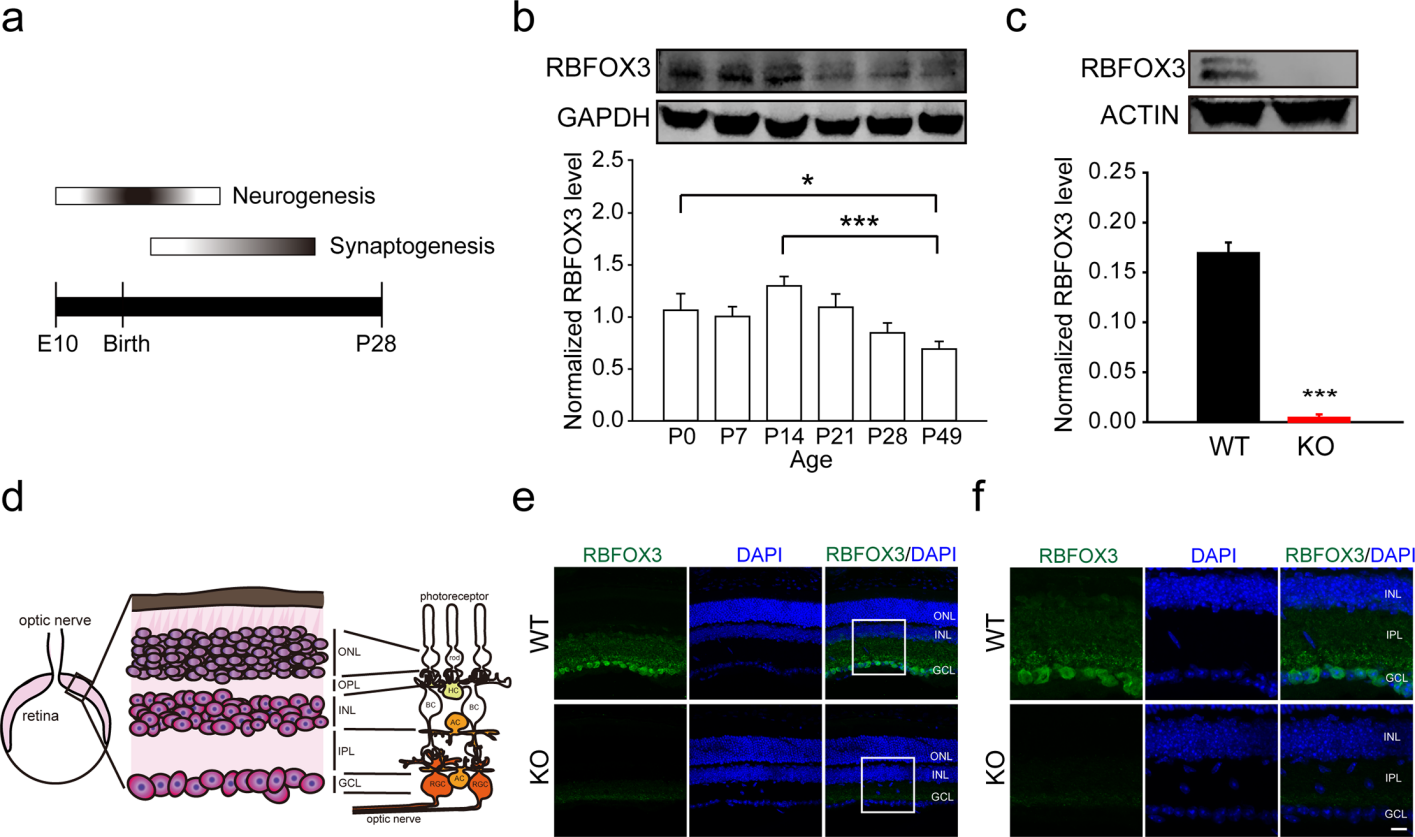
*Rbfox3* expression in retina is developmentally-regulated and layer-specific. (**a**) Schematic of developmental neurogenesis and synaptogenesis in the mouse retina [19, 22]. (**b**) Levels of RBFOX3 protein were measured from postnatal day 0 (P0) to 49 (P49) in the retina with western blot analysis using an antibody to NeuN, which recognizes RBFOX3, and were normalized to levels of GAPDH proteins. Kruskal-Wallis one-way ANOVA on ranks with Tukey *post hoc* comparison, *P < 0.05, ***P < 0.001. n = 12 per developmental time point. (**c**) Western blot analysis of retinal RBFOX3 protein from P49 wild-type (WT) and *Rbfox3* homozygous knockout (KO) mice. Levels of RBFOX3 proteins were normalized to levels of ACTIN proteins. Student’s t test, two-tailed, ***P < 0.001, n = 3 per group. (**d**) Left: Schematic of mouse retina. Middle: Schematic of retinal sublayers. ONL = outer nuclear layer; OPL = outer plexiform layer; INL = inner nuclear layer; IPL = inner plexiform layer; GCL = ganglion cell layer. Right: Schematic of cell subtypes located in each retinal layer. (**e**) Retinal regions from WT and KO mice immunostained with RBFOX3 antibody (green) and counter-stained with DAPI (blue). Scale bar = 20 μm. (**f**) Enlarged images of the white square in (**e**). Scale bar = 10 μm. All data are presented as mean ± s.e.m. All data points were available in S1 Table.

In mouse retina, neurogenesis occurs from embryonic stage to eleven days after birth [16–19]. Synaptogenesis takes place three days after birth and is completed three weeks after birth [20, 21] (Fig 1A). To investigate how *Rbfox3* expression is regulated during retinal development, we measured levels of RBFOX3 protein from postnatal day 0 (P0) to 49 (P49) in mouse retina with western blot analysis. Expression of retinal *Rbfox3* peaked at P14 and was down-regulated in the adult (*P* = 0.001, Kruskal-Wallis one-way ANOVA on ranks and *P* = 0.033 when P0 was compared to P49; *P* < 0.001 when P14 was compared to P49, Kruskal-Wallis one-way ANOVA on ranks with Tukey *post hoc* comparison) (Fig 1B and S1 Fig), which is similar to the developmental expression profiles of *Rbfox3* in other brain regions [3]. Our result suggests that retinal *Rbfox3* expression parallels the processes of retinal neurogenesis and maturation. To confirm whether *Rbfox3* was deleted in the retina of *Rbfox3* homozygous knockout (*Rbfox3*^*-/-*^) mice, we determined levels of RBFOX3 proteins in the retina of *Rbfox3*^*-/-*^ mice and their wild-type counterparts with western blot analysis. Two major isoform bands of RBFOX3 protein were detectable in WT mice, but were significantly reduced to barely detectable levels in the retina of *Rbfox3*^*-/-*^ mice (Fig 1C and S2 Fig), which has been demonstrated in other regions of mouse brain [3, 4].

**Fig 2:**
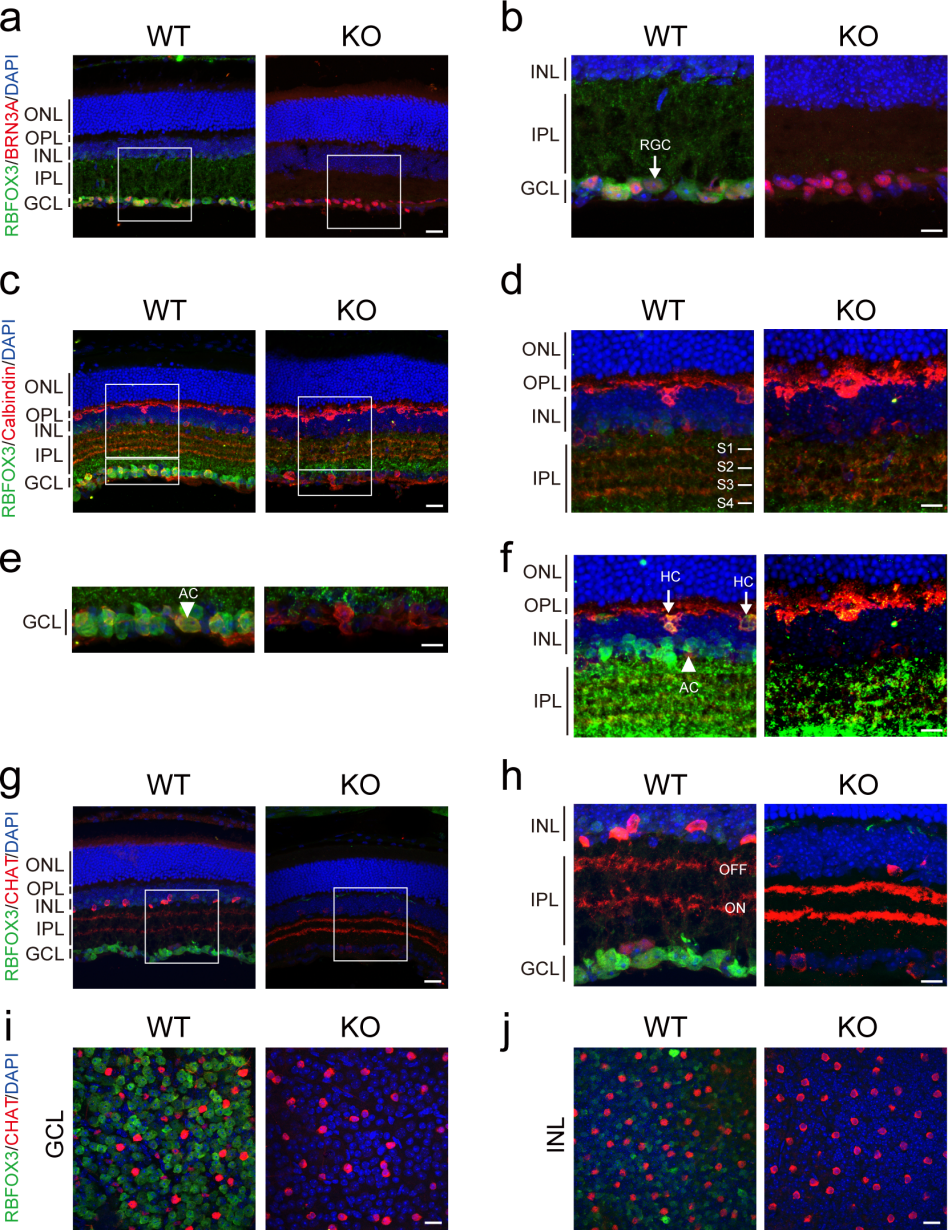
*Rbfox3* in the retina is exclusively expressed in ganglion cells, amacrine cells, and horizontal cells. Immunofluorescence staining was used to localize ganglion cells with their marker (BRN3A, red) (**a**), amacrine cells and horizontal cells with their marker (Calbindin, red) (**c**), and starburst amacrine cells with their marker (CHAT, red) (**g**) in the cross sections of retina of WT and KO mice. (**b**) Enlarged images of the white square in (**a**). Arrow indicates retinal ganglion cells. (**d**) Enlarged images of the large white square in (**c**). (**e**) Enlarged images of the small white square in (**c**). Arrow indicates AC.(**f**) Saturated exposure of FITC channel (green) from (**d**, WT and KO). Arrow indicates HC. (**h**) Enlarged images of the white square in (**g**). Whole-mount immunofluorescence staining was used to localize starburst amacrine cells with their marker (CHAT, red) in the GCL (**i**) and INL (**j**) of retina of WT and KO mice. Scale bar = 20 μm for (**a, c, g, i, j**). Scale bar = 10 μm for (**b, d, e, f, h**). Sections were stained with RBFOX3 (green) and counterstained with DAPI (blue). AC = amacrine cell. HC = horizontal cell.

Immunostaining of cross sections of the retina from wild-type mice showed that RBFOX3 is strongly expressed in the ganglion cell layer (GCL) but is weakly expressed in the inner and outer margin of the inner nuclear layer (INL) (Fig 1E and 1F, top), which is consistent with previous findings [10–12]. No signal for RBFOX3 protein was detected in the GCL or INL of *Rbfox3*^*-/-*^ mice (Fig 1E and 1F, bottom). Moreover, DAPI staining showed the thickness of the IPL was decreased in the retina of *Rbfox3*^*-/-*^ mice (Figs 1E, 1F and 2). Taken together, our results suggest retinal *Rbfox3* is developmentally regulated and is strongly expressed in the GCL but weakly expressed in the INL.

### Retinal *Rbfox3* is dominantly expressed in ganglion cells, amacrine cells and horizontal cells

**Fig 3:**
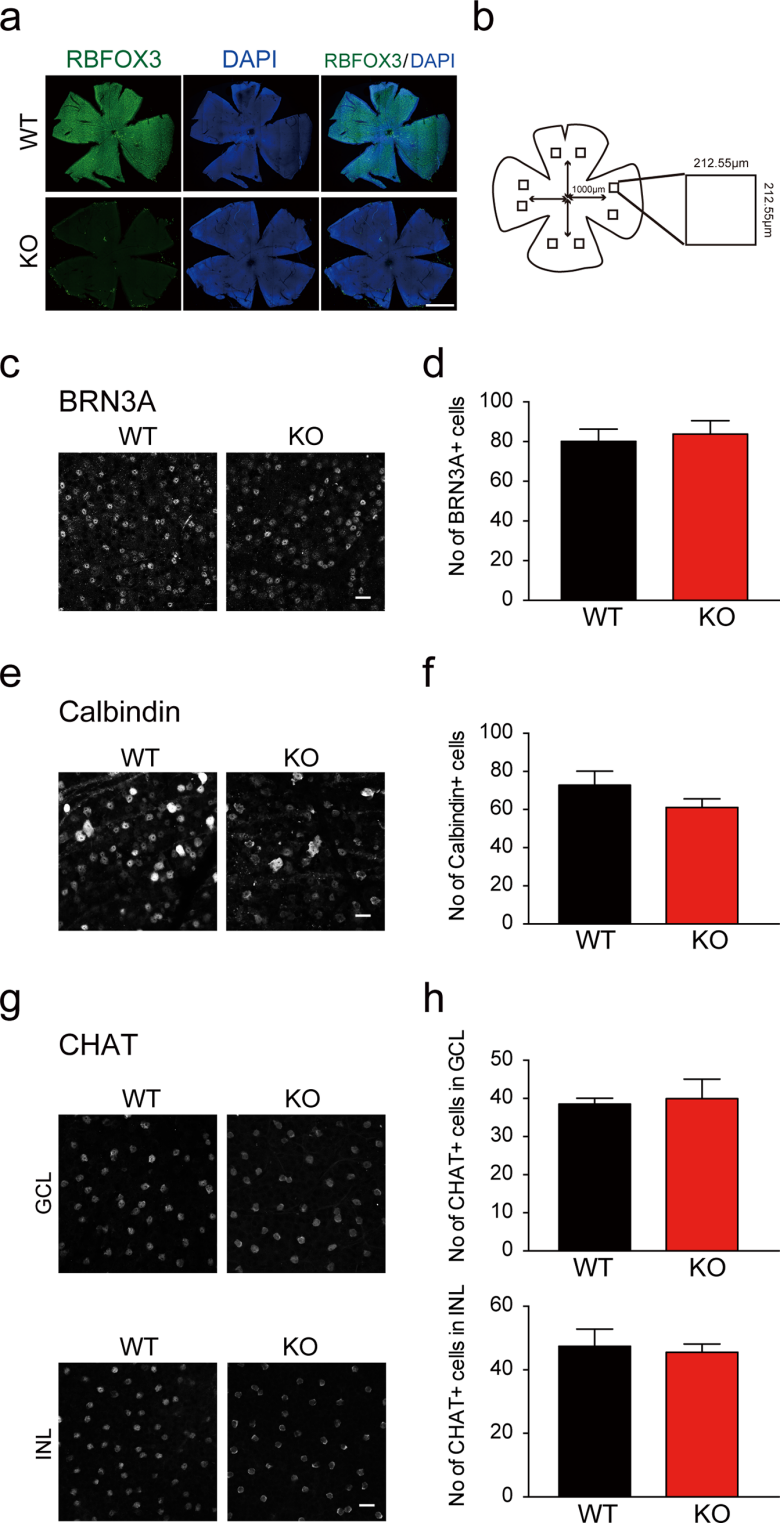
Loss of *Rbfox3* does not compromise the number of ganglion cells and amacrine cells in the retina. (a) Whole mount retinas from WT and KO mice immunostained with RBFOX3 antibody (green) and counter-stained with DAPI (blue). Scale bar = 1000 μm. (**b**) Schematic of four regions of mouse retina. Squares inside each region represent fields used for cell counts. Representative images and quantification in all four regions of retina in WT and KO mice: ganglion cells stained with anti-BRN3A antibody (**c**) and number of BRN3A-positive ganglion cells quantified (WT, n = 5 mice; KO, n = 5 mice) (**d**); amacrine cells stained with anti-Calbindin antibody (**e**) and number of Calbindin-positive amacrine cells quantified (Two-way ANOVA with Holm-Sidak *post hoc* comparison, *P < 0.05, **P < 0.01. WT, n = 4 mice; KO, n = 5 mice) (**f**); and anti-CHAT antibody with a focus on the GCL (upper panels) and INL (lower panels) (**g**) and quantification of ChAT-positive amacrine cells (WT, n = 3 mice; KO, n = 3 mice for both GCL and INL) (**h**). Scale bar = 20 μm. All data are presented as mean ± s.e.m. All data points were available in S1 Table.

The GCL of the retina contains ganglion cells and some displaced amacrine cells, whereas the majority of the INL consists of amacrine cells (Fig 1D). To further investigate which cell types specifically expressed RBFOX3 in the GCL and INL, we first performed immunofluorescence staining with anti-BRN3A antibody, an antibody which recognizes the majority of ganglion cells [23]. Although ganglion cells were present in the GCL of both wild-type and *Rbfox3*^*-/-*^ mice, RBFOX3 was only expressed in the ganglion cells of wild-type mice; no RBFOX3 signal was detected in the ganglion cells of the GCL in *Rbfox3*^*-/-*^ mice (Fig 2A and 2B). Calbindin has been used as a marker to label some displaced amacrine cells in the GCL, and some amacrine cells and horizontal cells in the INL of the retina [24]. Therefore, we applied anti-Calbindin antibody to detect those cell types in wild-type and *Rbfox3*^*-/-*^ mice (Fig 2C–2F). Our results showed that RBFOX3 was strongly expressed in amacrine cells in the GCL and weakly expressed in amacrine cells and horizontal cells in the INL in WT mice. No RBFOX3 signal was detected in such cell types in *Rbfox3*^*-/-*^ mice in the GCL or INL. Direction-selective ganglion cells (DSGCs) are connected post-synaptically to the starburst amacrine cells (SACs), which can specifically be labeled by anti-choline acetyltransferase (CHAT) antibody. We first labeled SACs in wild-type and *Rbfox3*^*-/-*^ mice with anti-CHAT antibody. Although CHAT labeled cells were present in the GCL and INL of wild-type mice (Figs 2G, 2H and 3G), RBFOX3 was not co-localized in the SACs of wild-type mice (Fig 2G and 2H). In addition, RBFOX3 signal was not detected in SACs of *Rbfox3*^*-/-*^ mice (Fig 2G and 2H). To confirm the absence of RBFOX3 in the SACs, we performed retinal whole-mount staining in wild-type and *Rbfox3*^*-/-*^ mice with anti-CHAT antibody. No signal for RBFOX3 was detected in the SACs (Fig 2I and 2J). Because the retina contains so many other types of amacrine cells, we also examined other subtypes of amacrine cells using markers for Calretinin and Tyrosine hydroxylase (TH). *Rbfox3* was expressed in both Calretinin- and TH-positive amacrine cells (S3 Fig). Moreover, about 70% of RBFOX3-positive cells are GCs and about 24% of RBFOX3-positive cells are ACs in the retinal GCL (S4 Fig). Taken together, our results suggest that retinal *Rbfox3* is expressed in most types of ganglion cells, some types of amacrine cells and horizontal cells.

### Loss of RBFOX3 does not alter numbers of BRN3A-positive ganglion cells, or Calbindin-positive and CHAT-positive amacrine cells

Knowing that RBFOX3 is correlated with retinal development and is expressed in the ganglion cells and amacrine cells of the retina, we investigated whether RBFOX3 is critical for retinal ganglion cells and amacrine cells. We quantified the number of BRN3A-positive ganglion cells and Calbindin-positive amacrine cells in the GCL of wild-type and *Rbfox3*^*-/-*^ mice with retinal whole-mount immunofluorescence staining (Fig 3A). We counted the number of labeled cells from two random fields of four regions (Fig 3B). No differences were detected between wild-type and *Rbfox3*^*-/-*^ mice in the number of BRN3A-positive cells in total four regions examined (Fig 3C and 3D). We also did not observe any difference between wild-type and *Rbfox3*^*-/-*^ mice in the number of Calbindin-positive cells (Fig 3E and 3F). Since RBFOX3 protein expression was almost non-detectable in SACs (Fig 2G–2J), we quantified the number of CHAT-positive SACs with whole-mount retinal staining to confirm the number of CHAT-positive SACs was not affected in *Rbfox3*^*-/-*^ mice. As expected, the number of CHAT-positive SACs were comparable between wild-type and *Rbfox3*^*-/-*^ mice in both the GCL and INL (Fig 3G and 3H). Our results indicate that RBFOX3 does not affect the number of ganglion cells and amacrine cells in the GCL.

### Deletion of *Rbfox3* causes a minor decrease in the thickness of the inner plexiform layer where synapses are formed

**Fig 4:**
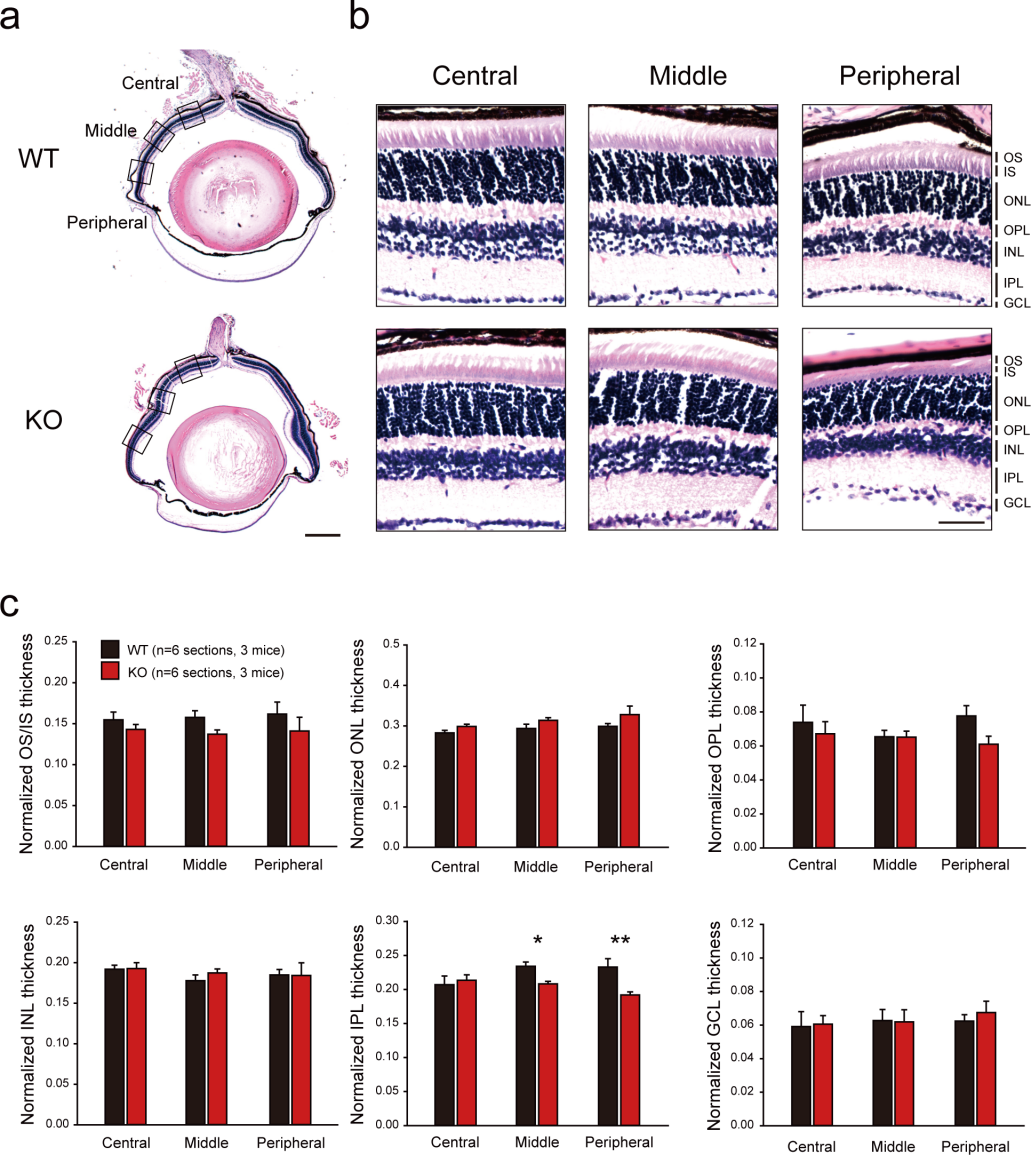
*Rbfox3* deletion decreased the thickness of the IPL in the retina. (**a**) Left: H&E staining of the whole eyeball of WT and KO mice. Scale bar = 500 μm. Squares in (**a**) are areas of the enlarged images for panel (**b**). Scale bar = 50 μm. (**c**) The thickness of each layer of different regions of the retina was quantified by normalizing to the total thickness of the retina. n = 3 mice for WT and KO group. Two-way ANOVA with Holm-Sidak *post hoc* comparison, *P < 0.05, **P < 0.01. All data are presented as the mean ± s.e.m. OS = outer segment; IS = inner segment; ONL = outer nuclear layer; OPL = outer plexiform layer; INL = inner nuclear layer; IPL = inner plexiform layer; GCL = ganglion cell layer. Middle = the area around the midpoint between the optic nerve head (ONH) and the edge of retina closest to cornea. Central = the area around the midpoint between ONH and “Middle”. Peripheral = the area around the midpoint between the “Middle” and the edge of retina closest to cornea. All data points were available in S1 Table.

To further investigate whether RBFOX3 is critical for the integrity of retinal morphology, we examined the thickness of each retinal layer from wild-type and *Rbfox3*^*-/-*^ mice with Hematoxylin and eosin (H&E) staining (Fig 4A). Due to the decline in thickness from the central to peripheral retina, we divided the retina into central, middle, and peripheral regions and measured the thickness of the layer per region (Fig 4A and 4B). When *Rbfox3*^*-/-*^ mice were compared with wild-type mice, the inner plexiform layer (IPL) in the *Rbfox3*^*-/-*^ mice exhibited a decreased thickness in the middle (*P* = 0.043, two-way ANOVA with Holm-Sidak *post hoc* comparison) and peripheral regions (*P* = 0.002, two-way ANOVA with Holm-Sidak *post hoc* comparison) (Fig 4C). The IPL contains synapses between axons of bipolar cells and amacrine cells in the INL and dendrites of ganglion cells in the GCL. Our results suggest that RBFOX3 could play a role in synapse formation in the IPL.

### Deletion of *Rbfox3* does not impair axon targeting of retinal ganglion cells

**Fig 5:**
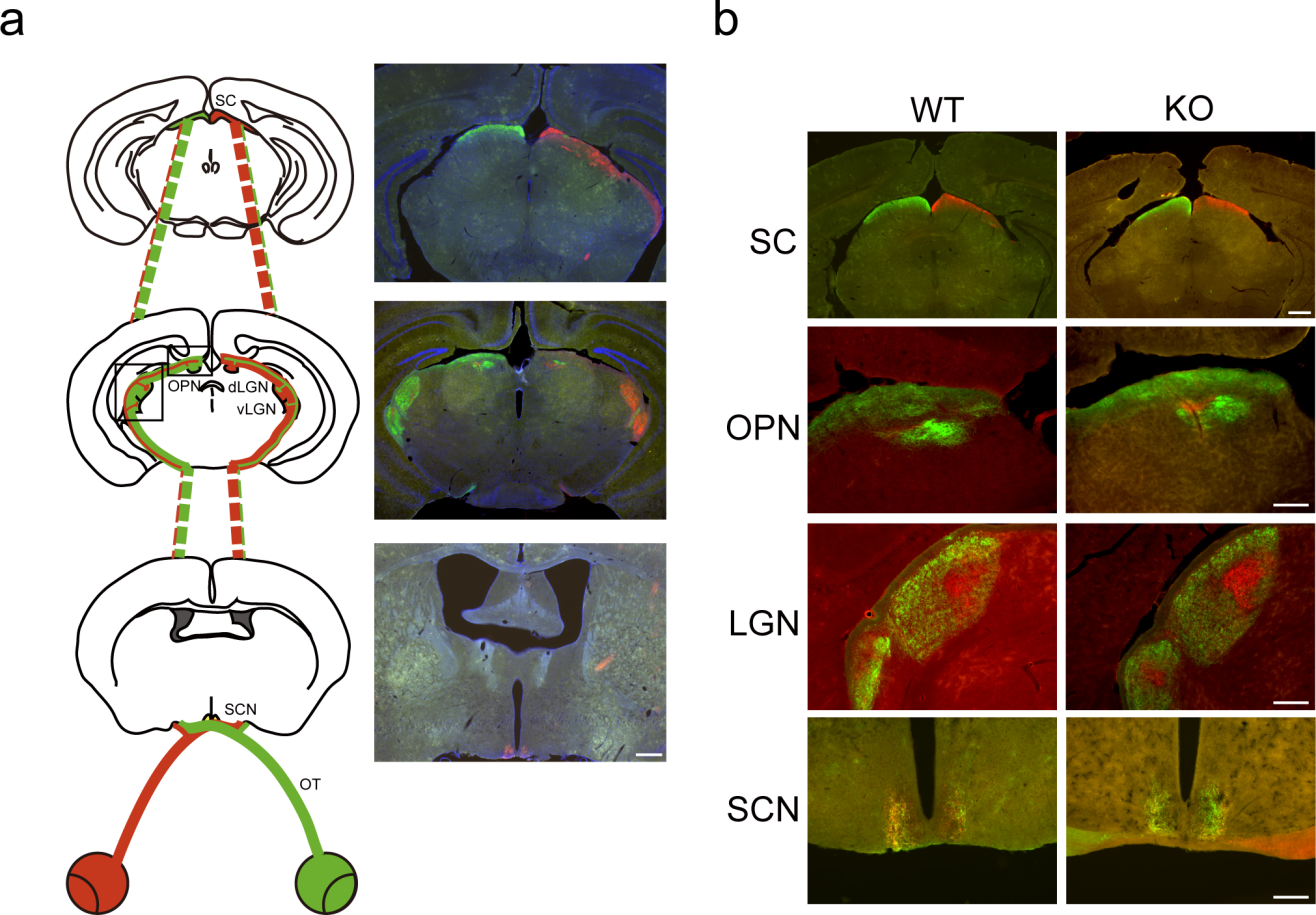
*Rbfox3* loss does not disrupt the innervation of retinal ganglion cells. (**a**) Left: Schematic of innervation of retinal ganglion cells into different brain regions following anterograde labeling using cholera toxin B subunit (CTB) conjugated with Alexa Flour 568 (red) injected into the right eye and CTB conjugated with Alexa Flour 488 (green) injected into the left eye. SC = superior colliculus. OPN = olivary pretectal nucleus. dLGN = dorsal lateral geniculate nucleus. vLGN = ventral lateral geniculate nucleus. SCN = suprachiasmatic nucleus. OT = optical tract. Right: Images of brain slices injected with CBT. Sections were counterstained with DAPI (blue). Scale bar = 500 μm. (**b**) CTB fluorescent signals on distinct brain regions from WT and KO mice. Scale bar = 500 μm for SC. Scale bar = 200 μm for OPN, LGN, and SCN.

*Rbfox3* is highly expressed in ganglion cells of the GCL. Therefore, we investigated whether RBFOX3 is required for axon targeting of retinal ganglion cells, which is critical for non-image and image forming function of the visual system. Cholera toxin B subunit (CTB) anterograde labeling was performed to examine four targeted brain regions for the axon terminals of retinal ganglion cells: the lateral geniculate nucleus (LGN), superior colliculus (SC), suprachiasmatic nucleus (SCN), and olivary pretectal nucleus (OPN) (Fig 5A). We observed that axons of retinal ganglion cells targeted the four brain regions correctly and normally in *Rbfox3*^*-/-*^ mice in comparison to their wild-type counterparts, regardless of brain hemisphere (Fig 5B). We also observed normal axon morphology of retinal ganglion cells in *Rbfox3*^*-/-*^ mice in comparison to their wild-type counterparts (S5 Fig). Our results suggest that RBFOX3 is not required for the projection of retinal ganglion cells axons to their targeted brain regions.

### RBFOX3 is not required for non-image and image forming function of the visual system

**Fig 6:**
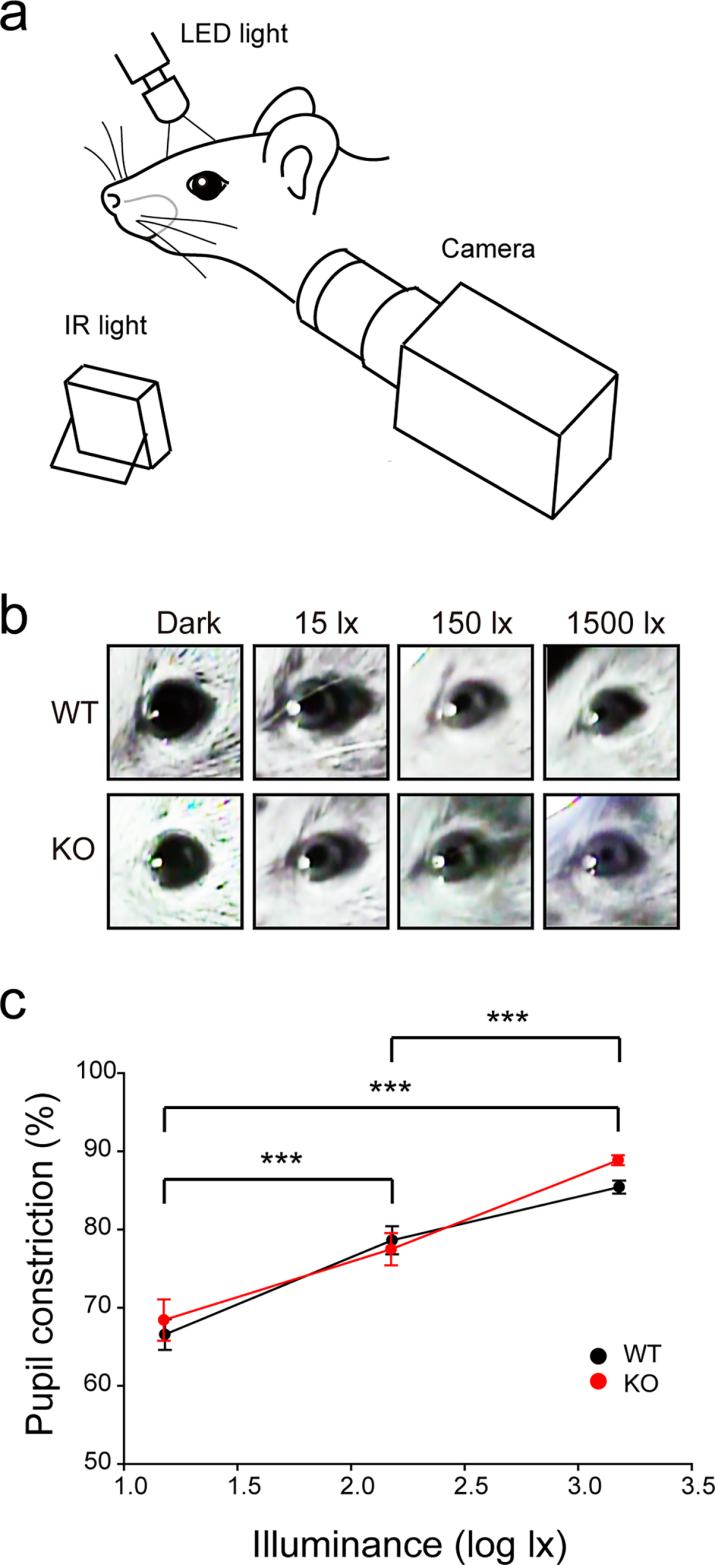
*Rbfox3* deletion does not affect the pupillary light reflex. (**a**) Schematic of the setup for pupillary light reflex experiments. (**b**) Representative pupil images from WT and KO mice under light conditions of dark, 15 lx, 150 lx, and 1,500 lx illuminance. (**c**) Size of pupil was quantified before and after light presentation from WT (n = 9) and KO (n = 6) mice. Two-way repeated ANOVA with Holm-Sidak *post hoc* comparison, ***P < 0.001. All data are presented as mean ± s.e.m. All data points were available in S1 Table.

To further investigate whether RBFOX3 affects visual function, we examined non-image and image forming function by measuring pupillary light reflex (PLR) and visual acuity, respectively in wild-type and *Rbfox3*^*-/-*^ mice. PLR is a reflex that controls the diameter of the pupil in response to the light that falls on the retinal ganglion cells. Specifically, the intrinsically photosensitive retinal ganglion cells (ipRGCs) are responsible for PLR. Moreover, PLR is a well-known non-image-forming visual response. We determined PLR (Fig 6A) and compared three different light intensities (15 lux (lx), 150 lx, and 1500 lx) with total darkness in wild-type and *Rbfox3*^*-/-*^ mice (Fig 6B). Under 15 lx light intensity, the percent of pupillary constriction was 66.56 ± 1.97% in wild-type mice and 68.54 ± 2.65% in *Rbfox3*^*-/-*^ mice; under 150 lx light intensity, the pupillary constriction was 78.64 ± 1.80% in wild-type mice and 77.62 ± 2.01% in *Rbfox3*^*-/-*^ mice; and under 1500 lx light intensity, the pupillary constriction was 85.44 ± 0.82% in wild-type mice and 89.01 ± 0.63% in *Rbfox3*^*-/-*^ mice (Fig 6C). A dosage-dependent effect of pupillary constriction in response to different light intensities was significant in both wild-type and *Rbfox3*^*-/-*^ mice. However, there was no difference in the percent of pupillary constriction under any light intensity between wild-type and *Rbfox3*^*-/-*^ mice (*P* = 0.443 under 15 lx, *P* = 0.694 under 150 lx, and *P* = 0.170 under 1500 lx, two-way repeated ANOVA with Holm-Sidak *post hoc* comparison) (Fig 6C). Our results suggest RBFOX3 is not required for the functional process of non-image forming function.

**Fig 7:**
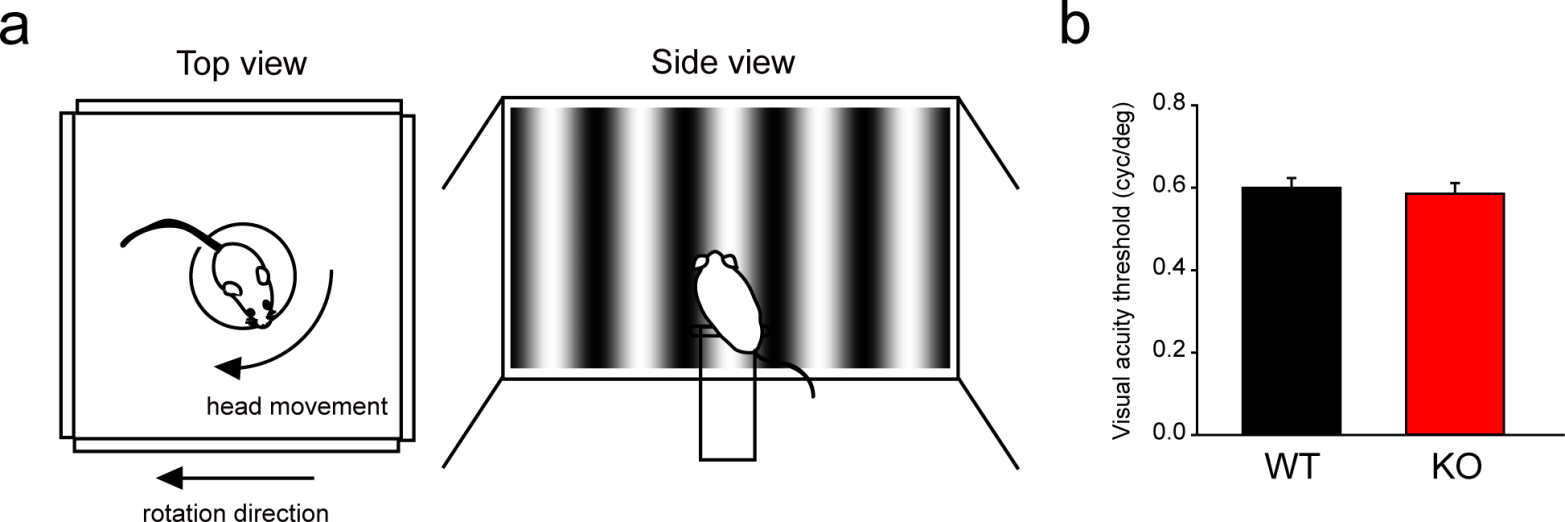
*Rbfox3* loss does not compromise visual acuity. (**a**) Schematic of top and side view of the optomotor tracking system. (**b**) Visual acuity thresholds were quantified from WT (n = 9) and KO (n = 7) mice. Mann-Whitney rank sum test, *P* = 0.723. All data are presented as mean ± s.e.m. All data points were available in S1 Table.

To measure the image forming function of *Rbfox3*^*-/-*^ mice, we performed an optomotor tracking response test and compared wild-type and *Rbfox3*^*-/-*^ mice to evaluate whether visual acuity was affected in *Rbfox3*^*-/-*^ mice (Fig 7A and S1 Movie). We assessed visual acuity by measuring the spatial frequency (vertical grating stimulus), which was set from 0.05 to 1.0 cyc/deg (Fig 7A). Head movements were used as responses to the spatial frequency in unrestrained mice. The visual acuity threshold was 0.60 ± 0.02 cyc/deg in wild-type mice and 0.59 ± 0.03 cyc/deg in *Rbfox3*^*-/-*^ mice, which was not significantly different (Fig 7B). Our results indicate that visual acuity is not altered in *Rbfox3*^*-/-*^ mice. Taken together, loss of RBFOX3 does not affect non-imaging or imaging forming visual function.

## Discussion

This is the first study to elucidate the role of RBFOX3 in the retina and visual function. We found that *Rbfox3* is developmentally regulated in the retina. Retinal RBFOX3 was demonstrated to be specifically expressed in the ganglion cells, amacrine cells and horizontal cells. Deletion of *Rbfox3* decreased the thickness of the inner plexiform layer, which is where synapses are formed between the axons of bipolar cells/amacrine cells and dendrites of the ganglion cells. Innervation of ganglion cells into their targeted brain regions was not significantly different between *Rbfox3*^*-/-*^ mice and their wild-type counterparts. In addition, RBFOX3 was not required for non-image and image forming function. Our results suggest that although loss of retinal RBFOX3 causes minor change of retinal morphology, it is dispensable for visual function.

Our study used an antibody to NeuN as an indicator of the presence of RBFOX3 protein. Researchers studying the retina frequently use anti-NeuN as a marker for ganglion cells [12, 25, 26]. Our results demonstrated that anti-NeuN stained not only ganglion cells (~ 70.40%) but also amacrine cells (~24.33%) in the GCL (S4 Fig). Therefore, we suggest anti-NeuN should be used with caution as a marker for ganglion cells. In addition, only 72.44% of cells in the GCL were immuno-reactive to the NeuN antibody (S6 Fig). One caveat of using mouse anti-RBFOX3 antibody (Millipore, MAB377) for immunofluorescence staining is that this antibody gets noise signals in the IPL especially in the central region of retinal sections (S6A Fig). We did not get such noise signals in the IPL when we used rabbit anti-RBFOX3 antibody (Millipore, ABN78) (S6B Fig).

Our previous findings showed that RBFOX3 is required for synaptic function and synaptogenesis in the hippocampal dentate gyrus [3, 4]. Our current results show *Rbfox3* is expressed exclusively in amacrine cells and ganglion cells of the retina. Interestingly, we found deletion of *Rbfox3* decreased the thickness of the inner plexiform layer, which is where synapses from the axons of bipolar cells and amacrine cells and dendrites of ganglion cells are formed. Synapses are also formed in the outer plexiform layer by the axons of photoreceptor cells from the outer nuclear layer and dendrites of horizontal cells/bipolar cells from the inner nuclear layer. Therefore, it is reasonable that we observed a decrease in the thickness of the inner plexiform layer of *Rbfox3*^*-/*-^ mice, but not in the outer plexiform layer. Since the number of retinal ganglion cell in *Rbfox3*^*-/*-^ mice is similar to their wild-type counterparts, our results suggest that decrease of synapse formation in the IPL could be the primary source for reduction in the thickness of the IPL. Surprisingly, although the thickness of the IPL is reduced in *Rbfox3*^*-/*-^ mice, the visual functions, including PLR and visual acuity, is normal. One explanation for this finding is the possibility that the global reduction of synapses does not impact the center-surround receptor field architecture which is essential for visual functions. This would explain the normal visual acuity of *Rbfox3*^*-/*-^ mice measured under our experimental setup. With regard to non-visual functions, ipRGCs could rely on the melanopsin photo-detection signaling pathway, thus a reduction in input from rods and cones might have limited impact on PLR. Finally, it is possible that the reduction in the thickness of the IPL is due to the loss of specific types of retinal neurons, which was not analyzed in this study. Determining the role of RBFOX3 in the formation or maintenance of IPL in the retina will require a more detailed examination of all cell types, which could be accomplished when more specific markers become available. We also can not rule out the role of RBFOX3 on the length or complexity of dendrites and axons in the IPL of the retina.

We previously showed that *Rbfox3*^*-/*-^ mice have normal motor function [3], however they have deficits in visual learning in the water maze test [4]. In this study, we examined visual function to determine if the deficit in visual learning was a result of a visual deficit. Our findings provide evidence that visual function in *Rbfox3*^*-/*-^ mice is not significantly different from their wild-type counterparts. One alternative explanation for the deficits in visual learning for *Rbfox3*^*-/*-^ mice in the water maze test may be a result of less motivation to swim (survive) or a possible attention-deficit like symptom. Different from measuring visual learning in the water maze test, the regular water maze measures the function of spatial learning. Our findings that *Rbfox3*^*-/-*^ mice do not have visual deficits, as measured by visual acuity and photo sensation, is further support that the poor performance of *Rbfox3*^*-/-*^ mice in the water maze test is a result of deficits in spatial learning [4]. This learning deficit in *Rbfox3*^*-/-*^ mice was also reproduced in the novel object recognition test, which assesses the function of learning/memory in mice [3]. The *Rbfox3*^*-/-*^ mouse model is important for studying human brain disorders associated with dysfunctional RBFOX3 [3, 4]. Our finding that *Rbfox3*^*-/-*^ mice possess normal visual function is important for the use of *Rbfox3*^*-/-*^ mice as a model for behavioral investigations.

Taken together, our findings show RBFOX3 may play a role in normal synaptic formation in the IPL of the retina, which is consistent with previous findings in the hippocampal dentate gyrus [3]. However, RBFOX3 is not required for development and maintenance of retinal ganglion cell number, innervation patterns of retinal ganglion cells, pupillary light reflex and visual acuity. Our results also rule out concerns regarding the potential visual confounding effects on behavioral experiments in *Rbfox3*^*-/-*^ mice [4].

## Supporting information

S1 Code The program of vertical grating stimuli. (PY)

S1 Fig Full-length blot for Fig 1B. (**a**) Original full-length blot. (**b**) High-contrast of full-length blot.
(TIF)

S2 Fig Full-length blot for Fig 1C. (**a**) Original full-length blot. (**b**) High-contrast of full-length blot.
(TIF)

S3 Fig *Rbfox3* in the retina is expressed in Calretinin- and TH-positive amacrine cells. Immunofluorescence staining was used to localize two subtypes of amacrine cells with their marker (Calretinin, red) (**a, top**), and (TH, red) (**b, top**) in the cross sections of retina of WT mice. Saturated exposure of Rhodamine channel (red) (**a, bottom**) and FITC channel (green) (**b, bottom**). Arrow indicates that amacrine cells co-localize with RBFOX3 and Calretinin or TH. Scale bar = 20 μm. Sections were stained with RBFOX3 (green) and counterstained with DAPI (blue).
(TIF)

S4 Fig Percentages of ganglion cells and amacrine cells in the RBFOX3-positive cells in the retinal ganglion cell layer. (**a**) RBFOX3-positive ganglion cells were identified by immunofluorescence staining with anti-RBFOX3 (green) and anti-BRN3A (red) antibodies. RBFOX3-positive amacrine cells were identified by immunofluorescence staining with anti-RBFOX3 (green) and anti-Calretinin (red) (**b**) or anti-Calbindin (red) (**c**) antibodies. Representative images were shown on the top of each panel. Pie charts indicate the percentages of ganglion cells and amacrine cells in the RBFOX3-positive cells. These charts were shown on the bottom of each panel. (**d**) Schematic of percentages of ganglion cells and amacrine cells in the RBFOX3-positive cells in the retinal ganglion cell layer. N = 20 sections, 4 mice for central, middle and peripheral regions of retinal sections. Sections were counterstained with DAPI. Scale bar = 20 μm. All data points were available in S1 Table.
(TIF)

S5 Fig *Rbfox3* deletion does not disrupt the axon morphology of retinal ganglion cells. Immunofluorescence staining was used to show axon morphology of retinal ganglion cells with an axon marker (neurofilament) in WT (**a, left**) and KO (**b, left**) mice. Enlarged images of central part of retina in WT (**a, right**) and KO (**b, right**) mice. Left scale bar = 500 μm; right scale bar = 200 μm.
(TIF)

S6 Fig Comparisons of immunofluorescence staining with two different anti-RBFOX3 antibodies. Immunofluorescence staining was performed with mouse anti-RBFOX3 (**a**, green) and rabbit anti-RBFOX3 (**b**, green) antibodies. The percentage of RBFOX3-positive cells in the retinal ganglion cell layer was calculated and demonstrated as pie charts (**a**, **b**, **bottom**). For mouse anti-RBFOX3 staining, n = 15 sections, 3 mice for central, middle and peripheral regions of retinal sections. For rabbit anti-RBFOX3 staining, n = 20 sections, 4 mice for central, middle and peripheral regions of retinal sections. Sections were counterstained with DAPI. Scale bar = 20 μm. All data points were available in S1 Table.
(TIF)

S1 Movie Videotaping of visual acuity test. (MOV)

S1 Table Summary of all data points in this manuscript. (XLSX)
