# Supplementary figures and images for: RBFOX3/NeuN is dispensable for visual function
Source: PLoS One. 2018 Feb 5;13(2):e0192355. doi: 10.1371/journal.pone.0192355 (PMC5798780; doi:10.1371/journal.pone.0192355)

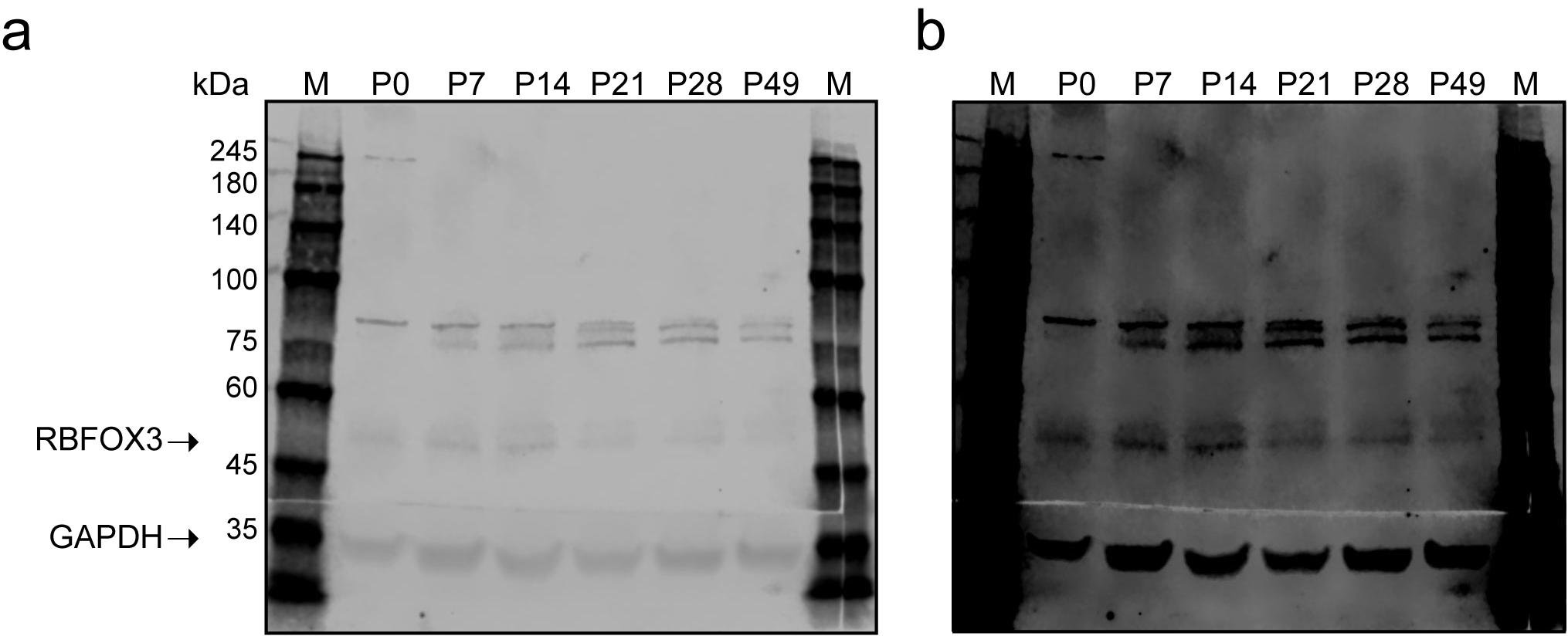

Supplement: S1 Fig — (a) Original full-length blot. (b) High-contrast of full-length blot. (TIF) [file pone.0192355.s002.tif]

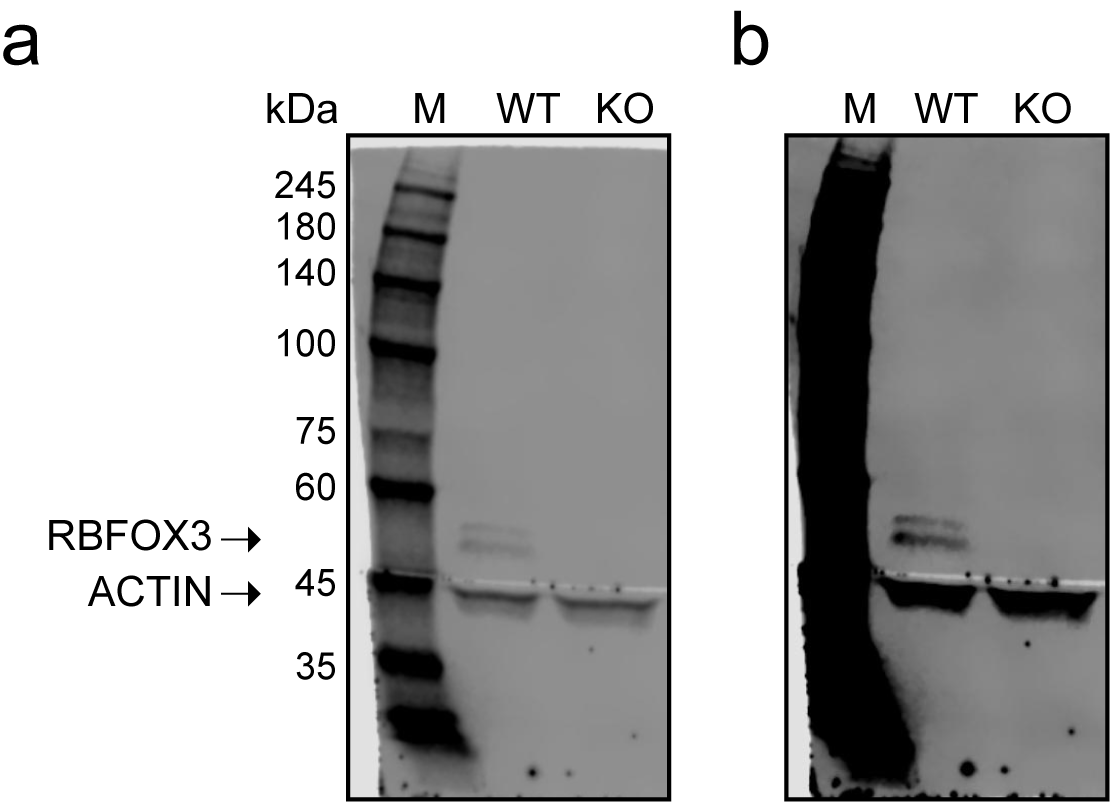

Supplement: S2 Fig — (a) Original full-length blot. (b) High-contrast of full-length blot. (TIF) [file pone.0192355.s003.tif]

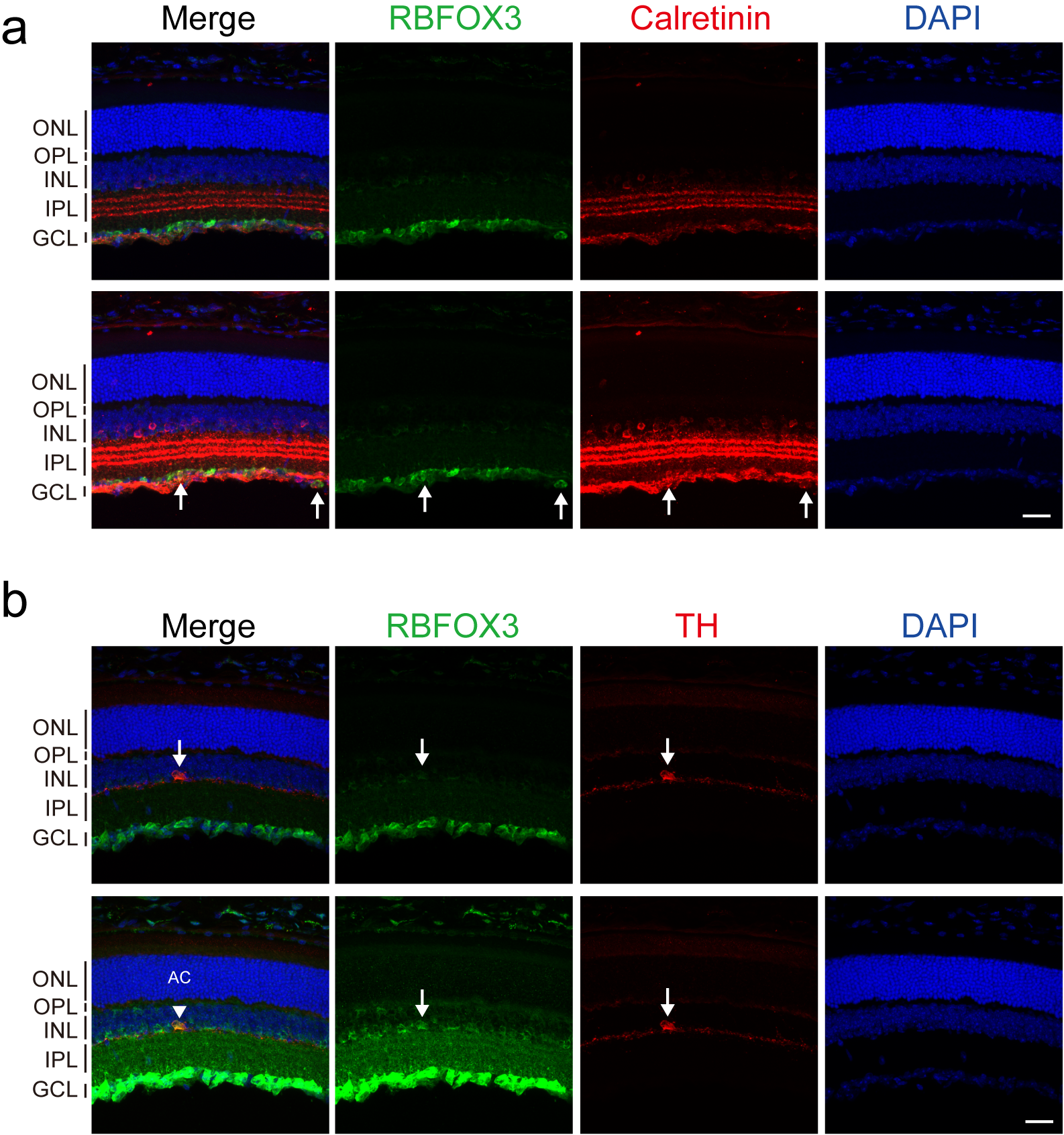

Supplement: S3 Fig — Immunofluorescence staining was used to localize two subtypes of amacrine cells with their marker (Calretinin, red) (a, top), and (TH, red) (b, top) in the cross sections of retina of WT mice. Saturated exposure of Rhodamine channel (red) (a, bottom) and FITC channel (green) (b, bottom). Arrow indicates that amacrine cells co-localize with RBFOX3 and Calretinin or TH. Scale bar = 20 μm. Sections were stained with RBFOX3 (green) and counterstained with DAPI (blue). (TIF) [file pone.0192355.s004.tif]

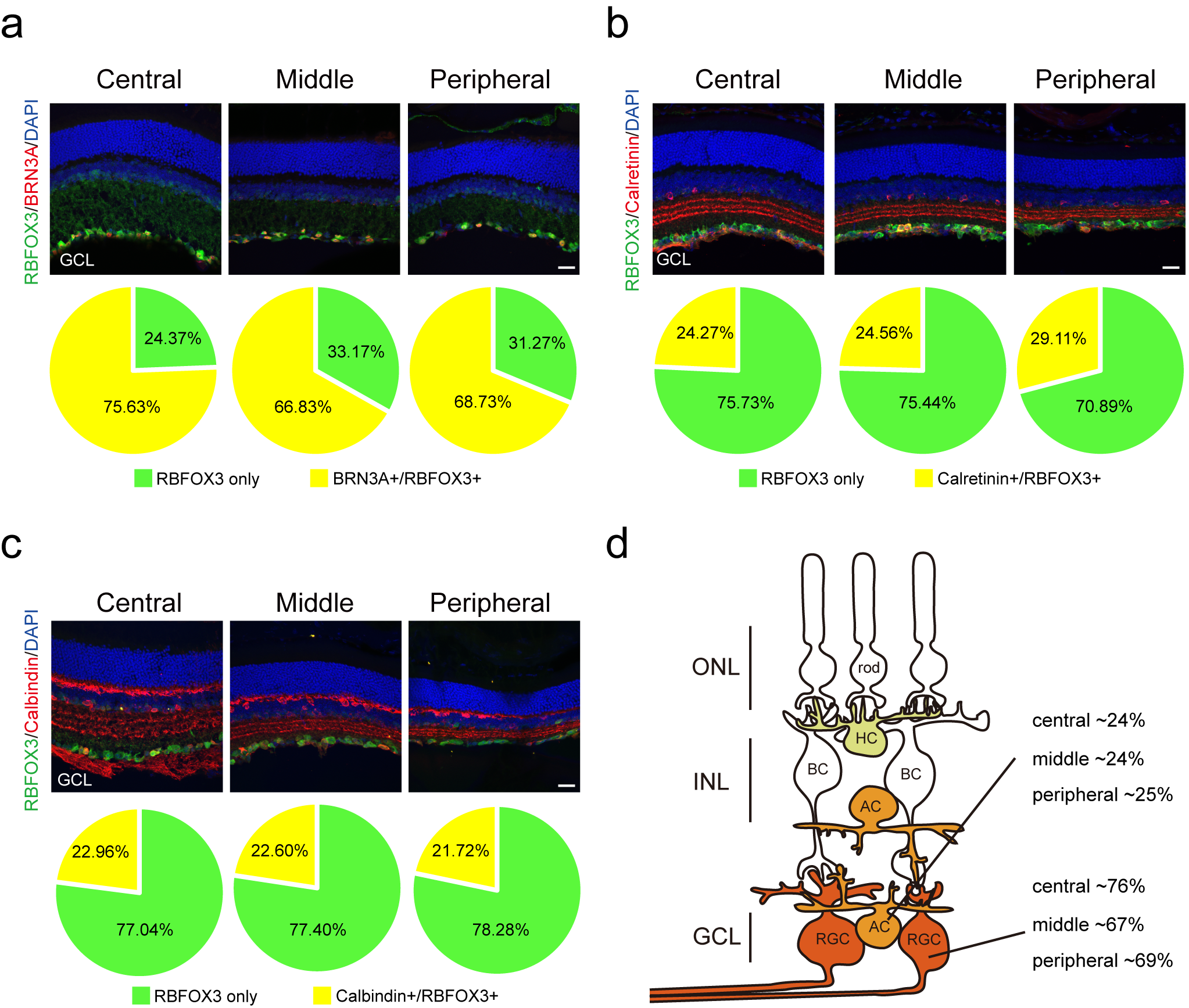

Supplement: S4 Fig — (a) RBFOX3-positive ganglion cells were identified by immunofluorescence staining with anti-RBFOX3 (green) and anti-BRN3A (red) antibodies. RBFOX3-positive amacrine cells were identified by immunofluorescence staining with anti-RBFOX3 (green) and anti-Calretinin (red) (b) or anti-Calbindin (red) (c) antibodies. Representative images were shown on the top of each panel. Pie charts indicate the percentages of ganglion cells and amacrine cells in the RBFOX3-positive cells. These charts were shown on the bottom of each panel. (d) Schematic of percentages of ganglion cells and amacrine cells in the RBFOX3-positive cells in the retinal ganglion cell layer. N = 20 sections, 4 mice for central, middle and peripheral regions of retinal sections. Sections were counterstained with DAPI. Scale bar = 20 μm. All data points were available in S1 Table. (TIF) [file pone.0192355.s005.tif]

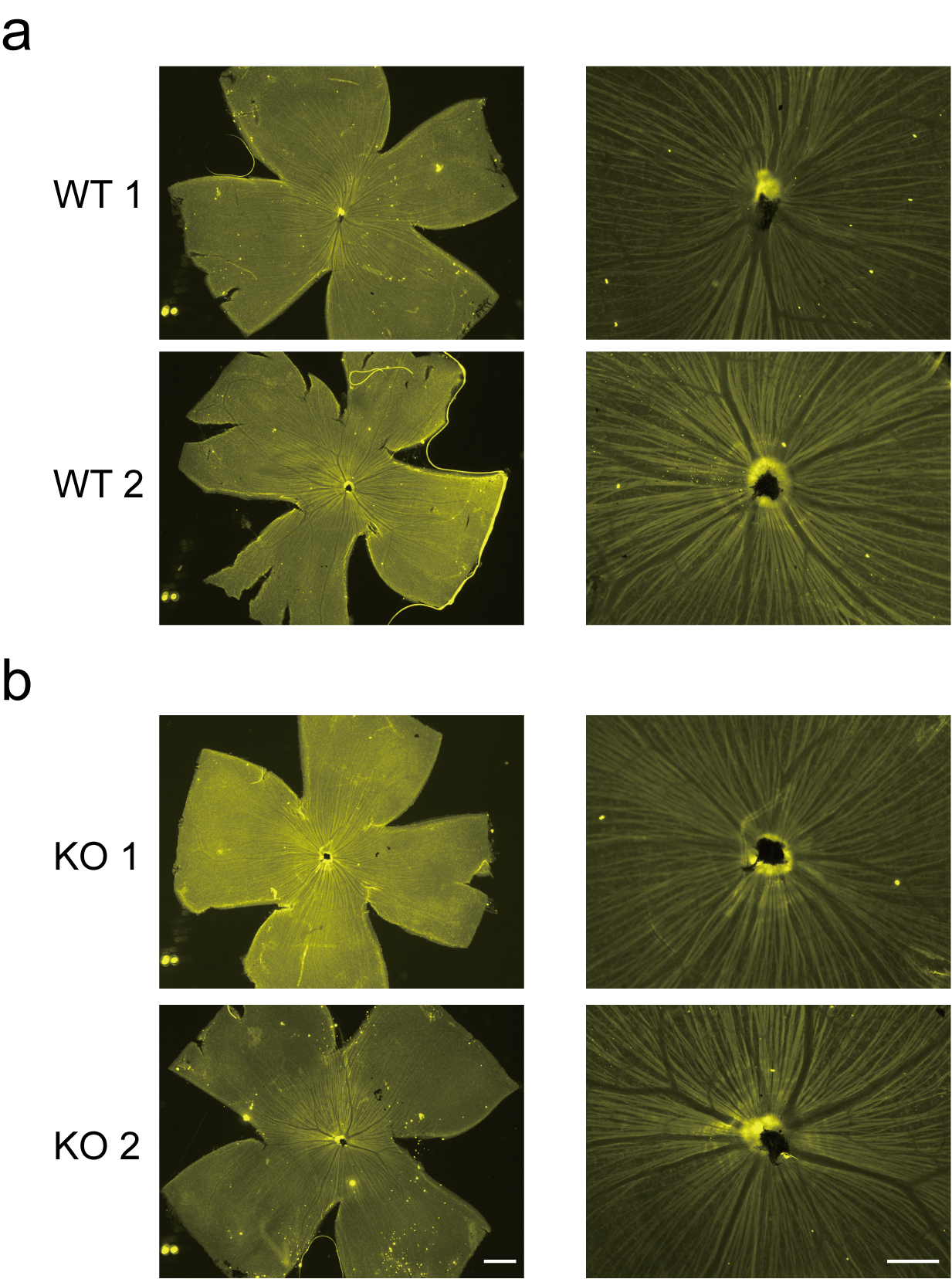

Supplement: S5 Fig — Immunofluorescence staining was used to show axon morphology of retinal ganglion cells with an axon marker (neurofilament) in WT (a, left) and KO (b, left) mice. Enlarged images of central part of retina in WT (a, right) and KO (b, right) mice. Left scale bar = 500 μm; right scale bar = 200 μm. (TIF) [file pone.0192355.s006.tif]

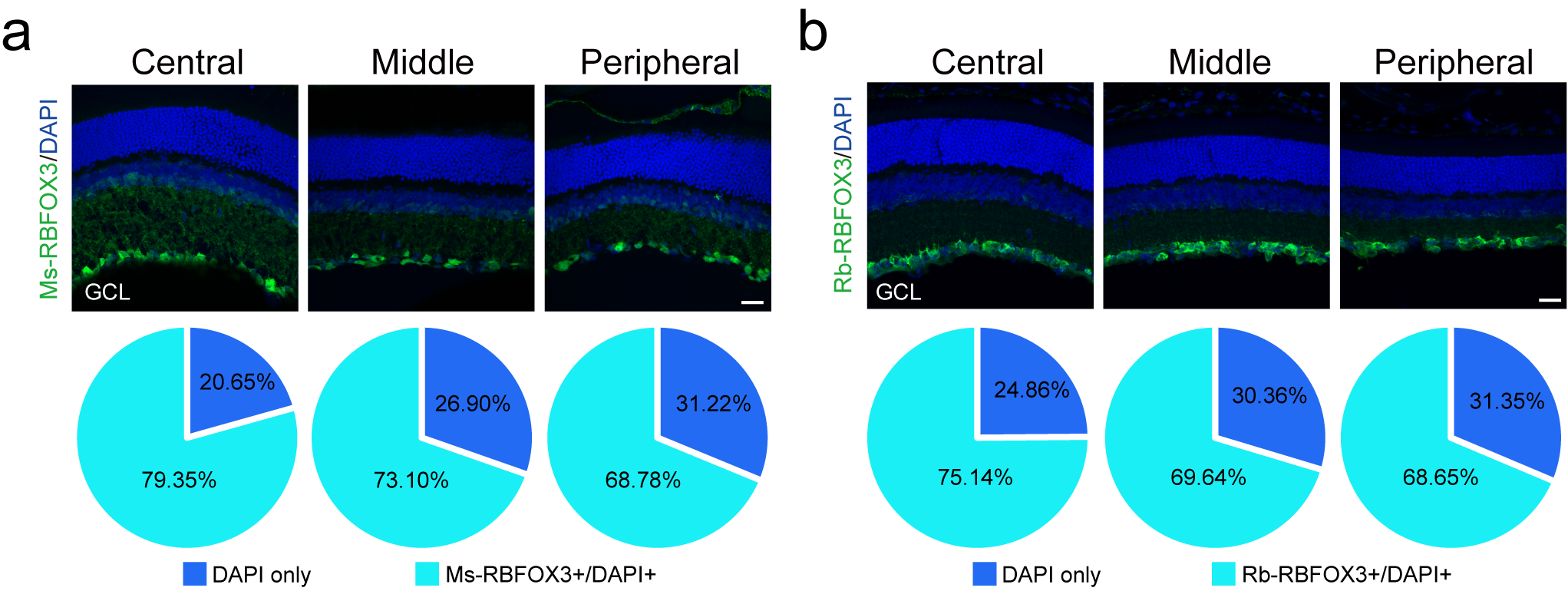

Supplement: S6 Fig — Immunofluorescence staining was performed with mouse anti-RBFOX3 (a, green) and rabbit anti-RBFOX3 (b, green) antibodies. The percentage of RBFOX3-positive cells in the retinal ganglion cell layer was calculated and demonstrated as pie charts (a, b, bottom). For mouse anti-RBFOX3 staining, n = 15 sections, 3 mice for central, middle and peripheral regions of retinal sections. For rabbit anti-RBFOX3 staining, n = 20 sections, 4 mice for central, middle and peripheral regions of retinal sections. Sections were counterstained with DAPI. Scale bar = 20 μm. All data points were available in S1 Table. (TIF) [file pone.0192355.s007.tif]
